# Photobiomodulation Therapy on the Guided Bone Regeneration Process in Defects Filled by Biphasic Calcium Phosphate Associated with Fibrin Biopolymer

**DOI:** 10.3390/molecules26040847

**Published:** 2021-02-05

**Authors:** Bruna Botteon Della Coletta, Thiago Borges Jacob, Luana Aparecida de Carvalho Moreira, Karina Torres Pomini, Daniela Vieira Buchaim, Rachel Gomes Eleutério, Eliana de Souza Bastos Mazuqueli Pereira, Domingos Donizeti Roque, Marcelie Priscila de Oliveira Rosso, João Vitor Tadashi Cosin Shindo, Marco Antônio Húngaro Duarte, Murilo Priori Alcalde, Rui Seabra Ferreira Júnior, Benedito Barraviera, Jefferson Aparecido Dias, Jesus Carlos Andreo, Rogério Leone Buchaim

**Affiliations:** 1 Department of Biological Sciences (Anatomy), Bauru School of Dentistry, University of São Paulo (USP), Bauru 17012-901, São Paulo, Brazil; brunacoletta@usp.br (B.B.D.C.); karinatorrespomini@gmail.com (K.T.P.); marcelierosso@usp.br (M.P.d.O.R.); jvshindo@gmail.com (J.V.T.C.S.); jcandreo@usp.br (J.C.A.); 2 Medical and Dentistry School, University of Marilia (UNIMAR), Marília 17525-902, São Paulo, Brazil; thiagoborges17@yahoo.com.br (T.B.J.); luana.cmoreira@bol.com.br (L.A.d.C.M.); danibuchaim@usp.br (D.V.B.); rachel.ge@hotmail.com (R.G.E.); elianabastosmsn@hotmail.com (E.d.S.B.M.P.); dune.roque@live.com (D.D.R.); 3 Postgraduate Program in Structural and Functional Interactions in Rehabilitation, University of Marilia (UNIMAR), Marília 17525-902, São Paulo, Brazil; jeff.bojador@gmail.com; 4 Medical School, University Center of Adamantina (UniFAI), Adamantina 17800-000, São Paulo, Brazil; 5 Center for the Study of Venoms and Venomous Animals (CEVAP), São Paulo State University (Univ Estadual Paulista, UNESP), Botucatu 18610-307, São Paulo, Brazil; rui.seabra@unesp.br (R.S.F.J.); bbviera@gmail.com (B.B.); 6 Department of Dentistry, Endodontics and Dental Materials, Bauru School of Dentistry, University of São Paulo (USP), Bauru 17012-901, São Paulo, Brazil; mhungaro@fob.usp.br; 7 Department of Health Science, Unisagrado University Center, Bauru 17011-160, São Paulo, Brazil; murilo.alcalde@usp.br; 8 Graduate Program in Tropical Diseases, Botucatu Medical School (FMB), São Paulo State University (UNESP – Univ Estadual Paulista), Botucatu 18618-687, São Paulo, Brazil; 9 Graduate Program in Clinical Research, Center for the Study of Venoms and Venomous Animals (CEVAP), São Paulo State University (UNESP–Univ Estadual Paulista), Botucatu 18610-307, São Paulo, Brazil; 10 Postgraduate Program in Law, University of Marilia (UNIMAR), Marília 17525-902, São Paulo, Brazil

**Keywords:** biomaterials, bone regeneration, fibrin biopolymer, low-level laser therapy, photobiomodulation therapy

## Abstract

The aim is to evaluate the effects of photobiomodulation therapy (PBMT) on the guided bone regeneration process (GBR) in defects in the calvaria of rats filled with biphasic calcium phosphate associated with fibrin biopolymer. Thirty male Wistar rats were randomly separated: BMG (*n =* 10), defects filled with biomaterial and covered by membrane; BFMG (*n =* 10), biomaterial and fibrin biopolymer covered by membrane; and BFMLG (*n =* 10), biomaterial and fibrin biopolymer covered by membrane and biostimulated with PBMT. The animals were euthanized at 14 and 42 days postoperatively. Microtomographically, in 42 days, there was more evident bone growth in the BFMLG, limited to the margins of the defect with permanence of the particles. Histomorphologically, an inflammatory infiltrate was observed, which regressed with the formation of mineralized bone tissue. In the quantification of bone tissue, all groups had a progressive increase in new bone tissue with a significant difference in which the BFMLG showed greater bone formation in both periods (10.12 ± 0.67 and 13.85 ± 0.54), followed by BFMG (7.35 ± 0.66 and 9.41 ± 0.84) and BMG (4.51 ± 0.44 and 7.11 ± 0.44). Picrosirius-red staining showed greater birefringence of collagen fibers in yellow-green color in the BFMLG, showing more advanced bone maturation. PBMT showed positive effects capable of improving and accelerating the guided bone regeneration process when associated with biphasic calcium phosphate and fibrin biopolymer.

## 1. Introduction

Bone has a high intrinsic capacity for regeneration as part of the repair process in response to injuries and in degenerative diseases, restoring its original structure and mechanical properties [1,2]. However, when the skeletal architecture is compromised, due to extensive bone defects, trauma, infections, tumor resection, skeletal abnormalities, avascular necrosis, and osteoporosis, bone regeneration becomes limited [3]. When bone repair is impaired or insufficient, reconstructive treatments are needed to assist the compromised physiological process, with autologous grafting being the main technique employed, due to its combined properties of osteogenesis, osteoinduction, and osteoconduction [4]. However, its use has become limited, due to the restricted bone quantity, the possibility of infections, and morbidity of the donor area [1,5].

In an attempt to replace the autogenous graft, alloplastic biomaterials have been a viable alternative, due to the possibility of optimizing their physical characteristics, obtaining materials with satisfactory osteoconductive properties [3,6]. Among alloplastics, biphasic calcium phosphate (BCP) and hydroxyapatite (HA) associated with β-tricalcium phosphate (β-TCP) are materials similar to the inorganic phase of bone tissue, providing excellent biocompatibility, atoxicity, which does not induce immunogenicity, biodegradability, and mechanical resistance, which makes the biomaterial favorable to new bone formation [7,8]. With the knowledge that biomaterials do not possess the properties of osteogenesis, osteoinduction, and osteoconduction, which are fundamental for the regenerative process, researchers have made an association between them in order to obtain synergistic effects. Thus, many studies have analyzed the incorporation of biomaterials into three-dimensional matrices, such as natural biopolymers, most commonly fibrin sealants [9,10,11].

Recently, the heterologous fibrin sealant has shown efficacy in the bone repair process, due to its three-dimensional structure, which allows the anchoring of cells and growth factors, which help in cell proliferation and differentiation. Thus, its name has been changed to fibrin biopolymer (FBP), due to its vast possibility of clinical applications [12,13,14]. In the search for the evolution of diverse regenerative techniques, new resources and treatment options have made it possible to achieve results close to the bone remodeling process. Thus, guided bone regeneration has become an effective technique in the treatment of bone defects. It is based on the concept of osteopromotion, with the objective of physically protecting the surgical site through membranes in order to prevent the invasion of cells coming from connective and epithelial tissue in situ, enabling bone neoformation [15,16].

Moreover, alternative non-invasive methods have been used in tissue regeneration, such as the use of low-level laser therapy (LLLT), currently called photobiomodulation therapy (PBMT) [17,18,19,20,21,22]. In the bone repair process, this therapy is known to exert analgesic anti-inflammatory effects, in addition to accelerating the regeneration process by providing cell proliferation and differentiation [23,24,25,26,27,28]. Thus, due to the low cost in relation to commercially available products, ease of acquisition, and biocompatibility that fibrin biopolymer and biphasic calcium phosphate present, studies that correlate biomaterials with PBMT in the guided bone regeneration process are necessary, assisting the grafting procedures that are routinely performed in medicine and dentistry with extensive bone loss.

Therefore, the objective is to evaluate the effects of photobiomodulation therapy (PBMT) in the guided bone regeneration process (GBR) in bone defects in the calvaria of rats filled with biphasic calcium phosphate associated with heterologous fibrin biopolymer.

## 2. Results

### 2.1. Microtomographic Analysis

**Figure 1:**
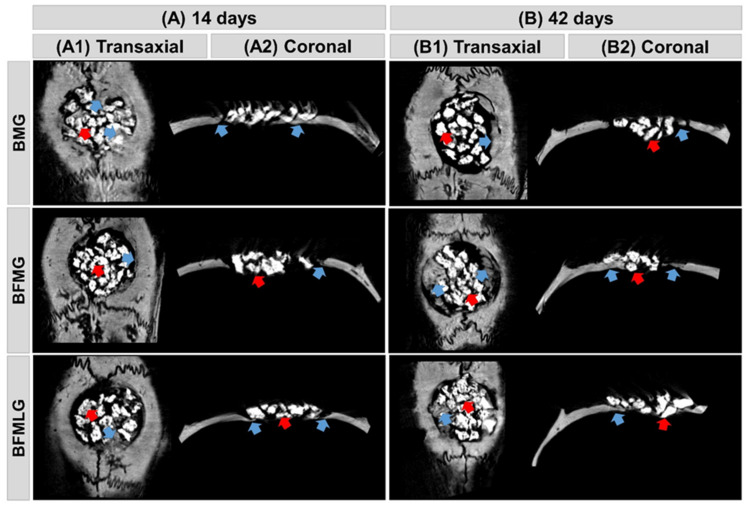
(**A**,**B**) Two-dimensional (2D) reconstructions of microtomographic images in transaxial (**A1**,**B1**) and coronal (**A2**,**B2**) sections of bone defects in the calvary of rats at 14 and 42 days, respectively. BMG—defects filled with biomaterial and covered by a membrane; BFMG—defects filled with the association of biomaterial and fibrin biopolymer, covered by a membrane; BFMLG—defects filled with the association of biomaterial and fibrin biopolymer, covered by a membrane and photobiomodulation therapy (PBMT). Blue arrow—new bone tissue; red arrow—particle of biomaterial.

Microtomographic images of all experimental groups revealed a gradual increase in radiopacity in a time-dependent manner and consistent with the centripetal bone growth, starting from the defect margins, evidenced by the hypodense areas of the newly formed tissue (see Figure 1A2,B2).

At 14 days, all bone defects were filled with radiopaque constituents referring to the particles of the biomaterial, without overlapping beyond the limits of the defect and permeated by hypodense areas of newly formed bone close to the defect edge (Figure 1A1,A2).

At 42 days, there was a progressive increase in density, represented by shades of gray, indicated by the new bone tissue, forming isodense areas, but without complete closure of the wound. At the end of the experimental period, the surgical defects were almost completely filled with particles, areas with no density are shown in black, and areas remodeled at the margins are most evident in the BFMLG group (Figure 1B1,B2).

### 2.2. Histomorphological Analysis

**Figure 2:**
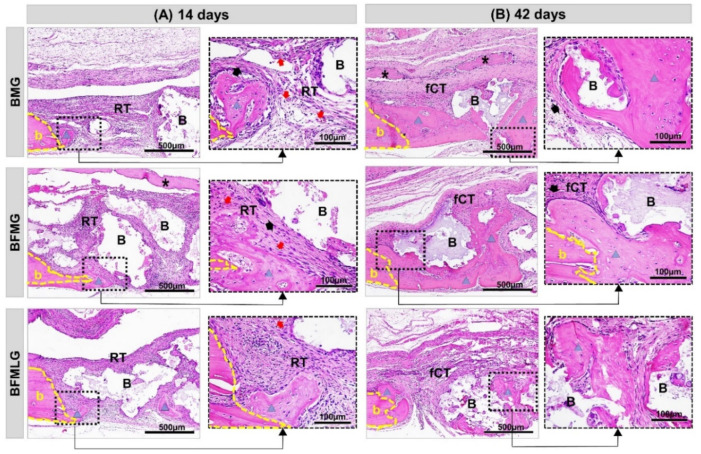
(**A**,**B**) Histological images of the evolution of defects in rat calvaria in the experimental periods of 14 and 42 days, respectively. BMG—defects filled with HA/β-TCP covered by a bovine biological membrane; BFMG defects filled with HA/β-TCP and heterologous fibrin biopolymer covered with bovine biological membrane; BFMLG defects filled with HA/β-TCP and heterologous fibrin biopolymer covered with bovine biological membrane and PBMT. Bone border (b), reactive connective tissue (RT), biomaterial particles (B), bovine biological membrane (asterisk), new bone tissue (blue triangle), fibrous connective tissue (fCT), collagen fibers (black arrow), blood vessel cells (red arrow). H.E. Original 10× magnification; bar = 500 µm and inserts, enlarged 40× images; bar = 100 µm).

**Figure 3:**
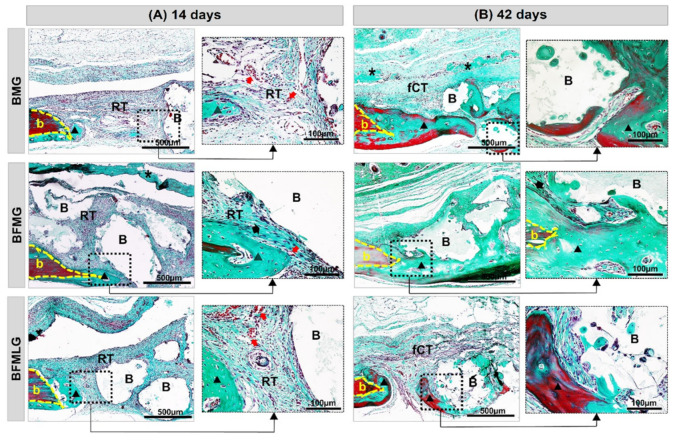
(**A**,**B**) Histological images of the evolution of defects in rat calvaria in the experimental periods of 14 and 42 days, respectively. BMG—defects filled with HA/β-TCP covered by a bovine biological membrane; BFMG defects filled with HA/β-TCP and heterologous fibrin biopolymer covered with bovine biological membrane; BFMLG defects filled with HA/β-TCP and heterologous fibrin biopolymer covered with bovine biological membrane and PBMT. Bone border (b), reactive connective tissue (RT), biomaterial particles (B), bovine biological membrane (asterisk), new bone tissue (black triangle), fibrous connective tissue (TCf), collagen fibers (black arrow), blood vessel cells (red arrow). Masson’s trichrome. Original 10× magnification; bar = 500 µm and inserts, 40× enlarged images; bar = 100 µm.

At 14 days, it was observed, in all experimental groups, well-defined bony edges with a flat chamfered shape and new immature bone tissue overlapping the dura mater. The entire surgical area was filled with biomaterial particles of various sizes surrounded by reactive connective tissue with the presence of inflammatory infiltrate and newly formed blood vessels (Figure 2A and Figure 3A).

In all animals, at 42 days, there was a slight increase in the formed bone tissue, with lamellar structure, surrounding the surfaces of the particles. The defects were interposed by a thin fibrous connective tissue with a reduction in the mononuclear inflammatory infiltrate and accompanied by a slight proliferation of blood capillaries. The collagen fibers were arranged circumferentially and parallel to the particles, which resulted in thick concentric layers. The membranes were not evident in all animals, but in some there were remnants on the surgical bed (Figure 2B and Figure 3B).

### 2.3. Histomorphometric Analysis

**Figure 4:**
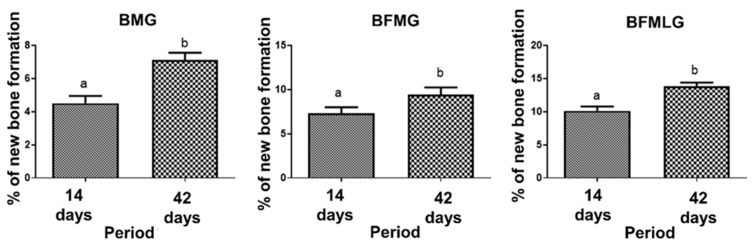
Percentage of new bone formation in each group (BMG, BFMG, and BFMLG) in the two experimental periods (14 and 42 days). Different lowercase letters indicate significant difference. Values defined as the mean ± standard deviation (*p* < 0.05), unpaired *t*-test.

A significant difference was observed in the percentage of new bone formation in all experimental groups when comparing the two periods of experimentation (14 and 42 days; Figure 4 and Table 1).

**Figure 5:**
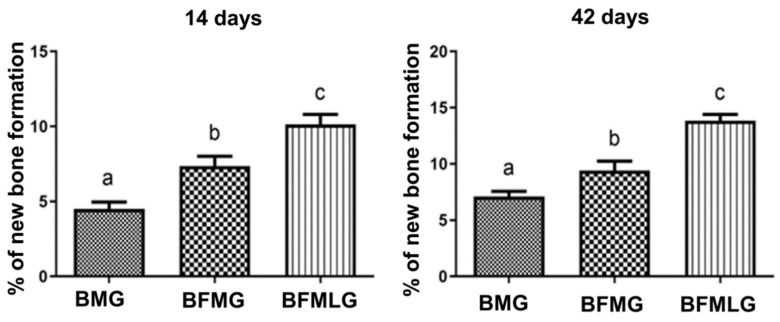
Percentage of new bone formation in each group (BMG, BFMG, and BFMLG) in the two experimental periods (14 or 42 days). Different lowercase letters indicate a significant difference. Values defined as the mean ± standard deviation (*p* < 0.05), ANOVA test for independent samples and the “post hoc” Tukey’s test (*p* < 0.0001).

When comparing the formation of new bone, in each of the experimental periods (14 or 42 days), a significant difference was observed between all groups analyzed (Figure 5 and Table 1).

### 2.4. Analysis of Birefringence of Collagen Fibers

Through the polarization of the collagen fibers stained by Picrosirius-red, it was possible to qualitatively analyze the level of bone maturation in the selected periods considering the quantity and thickness of the aligned filaments.

In this experimental protocol, the yellow-green birefringence color indicates thicker collagen fibers, lamellar bone, while the orange-red color indicates thin collagen fibers, immature bone, recognized for its random and disorganized fibrillar pattern.

**Figure 6:**
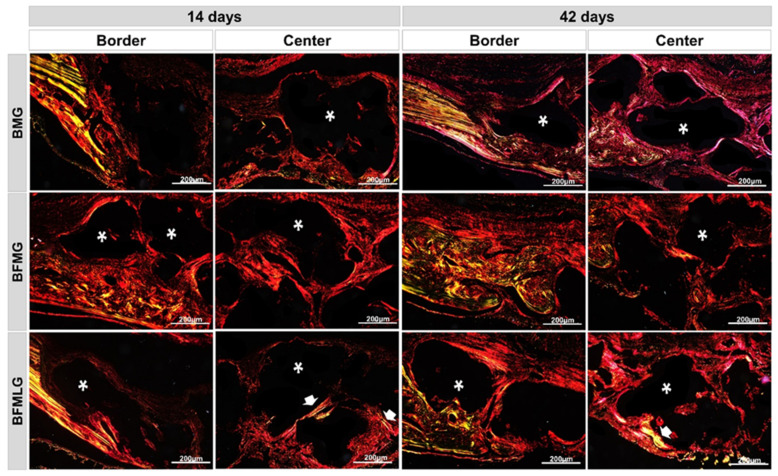
Histological sections of the border and center of the bone defect of rat calvaria stained by Picrosirius-red under polarized light at 14 and 42 days. BMG—defects filled with HA/β-TCP covered by a bovine biological membrane; BFMG defects filled with HA/β-TCP and heterologous fibrin biopolymer covered with bovine biological membrane; BFMLG defects filled with HA/β-TCP and heterologous fibrin biopolymer covered with bovine biological membrane and PBMT. RGB green-yellow-red colors. Mature bone—yellowish-green fibers (white arrow); immature bone—orange-red; particles of the biomaterial (dark background—asterisk). Original 10× magnification, 200 µm scale bar.

At 14 days, intense collagen synthesis was observed in all experimental groups, extending from the edges towards the center of the surgical defect, surrounding the biomaterial particles that remained on a dark background (Figure 6—see asterisks). In the BFMLG, regions in a more advanced stage of maturation were noted, with collagen fibers transitioning to yellowish-green birefringence more centrally (Figure 6—see white arrow).

At 42 days, all animals showed predominantly red birefringence and a greenish locus. However, the biostimulated animals (BFMLG) showed a higher intensity of birefringence of bright green collagen, indicating better the organization of type I collagen bundles (Figure 6).

## 3. Discussion

Due to the need for a treatment for the recovery of bone tissue when affected by critical size injuries, several techniques and materials have been investigated to facilitate and improve regeneration [5,29]. In light of this, the results of the present study showed that photobiomodulation therapy associated with biphasic calcium phosphate and fibrin biopolymer contributed to guided bone regeneration by defects in the calvaria of rats.

The protocol of this experiment was guided, in relation to the groups and the number of animals (n), in the principle of the 3 R’s (reduction, substitution, and refinement) in the use in scientific research. Therefore, it was decided not to carry out control groups with defects filled only by clot, biomaterial, or fibrin, which were widely published previously in the literature, including by the same group of researchers, focusing only on the need to recover extensive (critical) defects [26]. The critical size defect in research involving animals can be defined as a lesion size that does not heal for the total duration of the study, and in rats, it has a diameter of 5 mm [30].

Previous studies, performed by our research group, showed that the PBMT used, gallium-aluminum-arsenide (GaAlAs) with a wavelength of 830 nm, presented satisfactory performance, since it is able to penetrate deeper tissues such as bone, accelerating the tissue repair process [25,26]. However, due to the numerous dosimetry protocols, there is still no consensus in the literature as to the ideal application protocol [17,31]. Escudero et al. [21] demonstrated the effectiveness of PBMT in improving bone regeneration, reviewing 37 experiments and found several protocols, mainly with variations in wavelength and energy density, most of them (26 studies) using GaAlAs and 12 of them with 830 nm.

The wavelength used is within the therapeutic window, 820–840 nm, because in this spectral range, the more superficial chromophores of the intercalated layers have low selectivity, with greater penetration and consequently greater absorption by the chromophores and cytochrome-c oxidase that are present in the tissue bone [31,32,33]. The effects of PBMT are directly related to the wavelength and the loss of intensity that can compromise its function. Thus, the wavelength being in the infrared spectrum generates a small loss of intensity, which can reach 37% after a depth of 2 mm and, having the knowledge that the thickness of the integument in the surgical region of the rat calvaria presents small dimensions, in addition to the greater tissue penetration of the infrared laser, it generates the expectation that the loss, in this experimental protocol, is minimal [26,34].

PBMT, through the activation of irradiated tissue, has the potential to increase the formation of new bone, usually due to the following tissue events: direct action, stimulating the proliferation of osteoblasts, decreasing osteoclastic activity, favoring the production and maturation of collagen acting on fibroblasts, stimulating bone growth factors and modulating inflammatory cytokines; indirect action, creating a favorable microenvironment for the formation of bone tissue. These facts provide better bone mineralization and increase nitric oxide, which increases vascularity and, consequently, blood and lymphatic circulation [27,35].

In the microtomographic evaluation, it was possible to observe a progressive increase in new tissue formed, more evident in the BFMLG, with remodeling at the margin of the defect at the end of 42 days. This can be attributed to the exponential action of the PBMT throughout the experimental period, in the initial phase, 14 days, accelerating the inflammatory process, allowing the deposition of osteoid matrix and in the late phase, 42 days, leading to an increased expression of osteoblastic differentiation markers ALP, BMP-4, and RUNX_2_ [36]. Furthermore, all groups had areas with no density, black, until the end of the experiment, indicating that there was no complete bone regeneration because it is a critical size lesion according to Gosain et al. [37].

Histologically, at 14 days, the presence of reactive connective tissue with inflammatory infiltrate and newly formed blood vessels was noted in all experimental groups. This inflammation can be explained that lesion causes, changes in the immunogenic characteristics of the cells with the implantation of biomaterials as a protective response, are of great importance for the communication of osteogenic cells with inflammatory cells in the initial remodeling process [38,39,40].

At 42 days, it is possible to notice a slight tissue growth with controlled inflammatory response and collagen fibers arranged in concentric layers, indicating secondary bone tissue. It is suggested that this scenario is related to the biocompatibility and osteoconductive capacity of biomaterials. This is because biphasic bioceramics have hydroxyapatite in their composition, which is similar to the inorganic phase of bone tissue, in addition to presenting macropores of different sizes that favor cell migration and restocking [41,42,43].

Allied to biphasic ceramics, the fibrin biopolymer (FBP), acting as a scaffold, facilitated the insertion of the graft. Due to its structure, it promoted greater stability at the defect site [14,44,45]. Such findings are in line with studies that used FBP for the same purpose, obtaining favorable results in tissue regeneration [12,46]. The proportion of its components and the sequence of manipulation is adjusted according to the purpose of its use, for example, in the repair of nerve injuries [47], bone [14,48,49], venous ulcers [50,51], and stem cells [46,52,53].

The bovine cortical bone membrane was used in all groups in order to act as a physical barrier, preventing non-osteogenic cells from invading the surgical area [54,55,56]. Until the end of the experimental protocol, it was possible to observe remnants of the membrane.

In the histomorphometric analysis, there was a significant difference in all groups, which showed bone growth from 14 to 42 days, confirmed qualitatively by microtomographic analysis. Comparing groups within the same experimental period, the BFMLG showed greater bone formation in both periods (10.12 ± 0.67 and 13.85 ± 0.54), followed by BFMG (7.35 ± 0.66 and 9.41 ± 0.84) and BMG (4.51 ± 0.44 and 7.11 ± 0.44). This can be attributed to factors such as the PBMT irradiation protocol used, which has been tested in our research group, showing positive effects on tissue regeneration [24,25,26,57].

Moreover, a synergistic effect of fibrin biopolymer with bioceramics can be considered. The combination of two scaffolds, one of fibrin, as the heterologous biopolymer, and the other, an alloplastic biomaterial, showed in this experiment effectiveness in the structural and functional properties in the formation of new bone. The three-dimensionality of the structure of fibrin derivatives with the porosity of the biomaterial provides an ideal microenvironment for the differentiation and growth of bone precursor cells and neovascularization, this association being a promising alternative in tissue bioengineering [14,46,58]. It is important to show that, although GenPhos XP^®^ is already commercialized and used widely in dentistry, no studies were found involving this biomaterial to the fibrin biopolymer, listing the present research as a pioneer in the process of guided bone regeneration in the calvaria of rats.

The registration of collagen fibers can be considered an important indicator of bone formation, with type I collagen being the main component of the organic matrix of the bone, responsible for the flexibility of the bone [59,60]. According to our qualitative birefringence data, at 14 days, it was possible to observe an intense collagen synthesis in all groups. Such data may be due to PBMT in the differentiation and proliferation of fibroblastic cells. Studies demonstrate an increase in the differentiation of fibroblasts with PBMT, including myofibroblasts, an important fact for the repair of lesions, regardless of the TGF-β1/Smad pathway, which acts in the inhibition or stimulation of cell growth. PBMT has the potential to stimulate the proliferation and migration of fibroblasts, which are essential for reepithelization, angiogenesis, and formation of granulation tissue and, consequently, stimulate repair [61,62]. 

The BFMLG stood out from the others for presenting regions in an advanced stage of maturation with a transition from the birefringence color of the yellowish-green collagen fibers [26]. At 42 days, the BFMLG was also the group that showed the best organized pattern of bundles of type I collagen, evidenced in bright green color, characterizing the lamellar bone. It is suggested that PBMT may have contributed to the parallel arrangement and thickening of collagen fibers, providing the deposit of inorganic salts for the formation of mature bone. In previous studies with PBMT and bone repair [63,64], the Picrosirius-polarization method is a useful color to correlate the organization of collagen fibers with the development of lamellar bone, detecting collagen types I, II, and III, as a possible indicator of the aggregation of collagen molecules. 

This fact shows that PBMT stimulates the growth of the trabecular area and the simultaneous invasion of osteoclasts in the initial periods and accelerates the organization of the matrix collagen (parallel alignment of fibers) at a more advanced stage [65]. Understanding the spatial organization and the composition of the collagen fibers that, from their calcification, will form the lamellae is important for understanding the physiological, phytogenetic, mechanical, and ecological aspects of bone formation [66].

For prospective studies, the use of other physical therapies are suggested that assist bone growth such as low-intensity pulsed ultrasound (LIPUS) [67] and LED phototherapy [68]. In addition, PBMT with Laserpulse Ibramed^®^ [69], GenDerm^®^ bovine bone membrane [70], and the biomaterial GenPhos XP^®^ [71] have been used clinically in several health areas, mainly in dentistry and regenerative medicine. The fibrin biopolymer is in phase II/III of clinical tests at the Brazilian Health Regulatory Agency (ANVISA) in the treatment of venous ulcers, and pre-clinical tests in several areas have demonstrated its translational potential [45,51,72].

## 4. Materials and Methods

### 4.1. Alloplastic Biomaterial

The biomaterial used was the GenPhos XP^®^ (Baumer S.A., Mogi Mirim, São Paulo, Brazil, Ministry of Health Registry No. 10345500076). It is a biphasic bioceramic composed of hydroxyapatite and β-tricalcium phosphate in the proportion of 70–30% respectively, granulometry between 0.50 to 0.75 mm and packed in 0.7 g packaging. It presents an irregular surface with several concavities and cracks, in addition to macros and micropores of different diameters with an estimated resorption time between 7 to 9 months. GenPhos XP^®^ is used in implant dentistry and maxillofacial and bone surgery procedures and can be considered a safe alternative to autologous grafting [43,73].

### 4.2. Fibrin Biopolymer

The fibrin biopolymer was provided by the Center for the Study of Venoms and Venomous Animals—CEVAP of the São Paulo State University “Júlio de Mesquita Filho”, registration n° BR1020140114327 and BR1020140114360. The package available for clinical research consists of three solutions, packaged separately, and kept at −18 to −22 °C.

- Fraction 1: thrombin-like or gyroxin purified from the poison of *Crotalus durissus terrificus*, 0.4 mL.
- Diluent: calcium chloride, 0.6 mL.
- Fraction 2: fibrinogen, cryoprecipitate derived from the blood of buffalo (*Bubalus bubalis*), 1 mL.

The proportion used (1:1:2) was in accordance with the manufacturer’s recommendations and the amount was readjusted through a pilot study.

#### Preparation of the Biopolymer

The solutions were thawed and kept at room temperature between 15–30 °C and then homogenized. In BFMG and BFMLG animals, 10 µL of fraction 1 and 10 µL of diluent were added to 0.015 g of biomaterial, and then the mixture was incorporated to 20 µL of fraction 2 for polymerization to occur.

### 4.3. Guided Bone Regeneration (GBR)

The membrane used to perform the GBR was the GenDerm^®^ (Baumer S.A., Mogi Mirim, São Paulo, Brazil, with registration number 10345500069), derived from bovine cortical bone, with flexible and resorbable characteristics. Its manufacturing process provides a porous, acellular, biocompatible, non-antigenic, pyrogen-free, high-purity membrane, free from contamination with heavy metals and other proteins. The choice to use the GenDerm^®^ membrane is supported in the literature with its use in bone defects such as calvaria and tibia of rats to prevent the invasion of soft tissues at the injury site and assist in regeneration [55,74,75]. For this experiment, the membrane was used with dimensions of 20 × 20 mm (height/width), with size adaptation according to the defect made in the present study.

### 4.4. Experimental Design

Thirty male adult Wistar rats (*Rattus norvegicus*) were used, weighing approximately 250 g, provided by the Central Bioterium of the University of Marília after approval by the Animal Use Ethics Committee (CEUA) of the University of Marília under protocol 04/2018, number CIAEP-01.0218.2014, of 28 June 2018.

The animals were kept in conventional cages containing 04 animals each, under artificial lighting controlled by a timer, a 12-h light/dark cycle, an exhaust fan, and air conditioning to maintain an average temperature around 22 °C.

**Figure 7:**
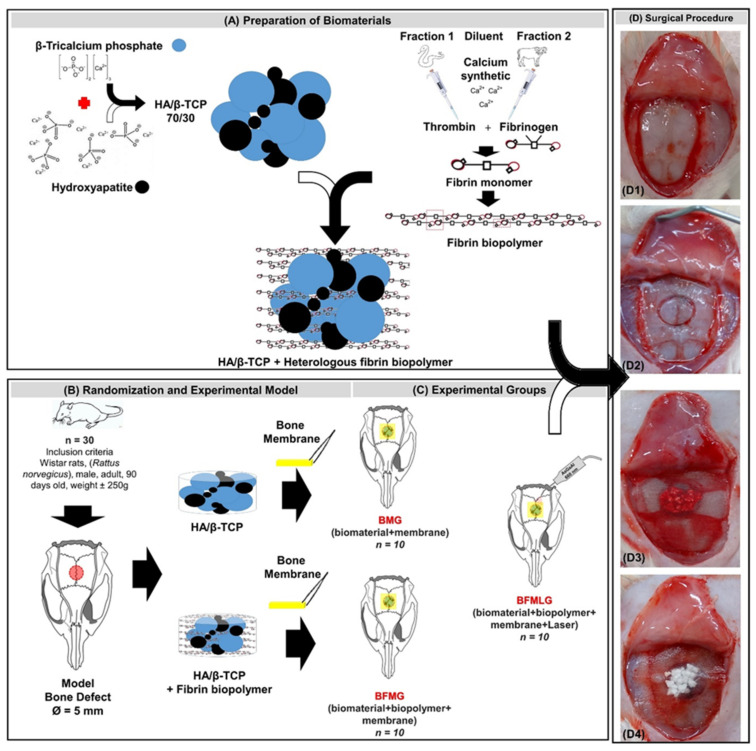
Schematic drawing of the experimental design. (**A**) Preparation of biomaterials: alloplastic hydroxyapatite and beta tricalcium triphosphate in the ratio of 70:30 (HA/β-TCP 70/30). Heterologous fibrin biopolymer—fraction 1 (thrombin-like or gyroxin), diluent (synthetic calcium), fraction 2 (buffalo fibrinogen). HA/β-TCP association and heterologous fibrin biopolymer. (**B**) Randomization and experimental model: 30 male adult Wistar rats. Experimental model—5 mm bone defect in the center of the parietal bones. (**C**) Experimental groups: BMG (*n* = 10), defects filled with HA/β-TCP and covered by a membrane; BFMG (*n* = 10), defects filled with the association of HA/β-TCP and fibrin biopolymer, covered by a membrane; BFMLG (*n* = 10), defects filled with the association of HA/β-TCP and fibrin biopolymer, covered by a membrane and PBMT. (**D**) Surgical procedure: (**D1**) Parietal exposure. (**D2**) Defect in the center of the parietal bones. (**D3**) Defect filled with HA/β-TCP and heterologous fibrin biopolymer. (**D4**) Bone defect filled with HA/β-TCP and heterologous fibrin biopolymer covered by a membrane derived from the bovine bone cortex.

The animals were randomly separated into three groups: BMG (*n* = 10), defects filled with biomaterial and covered by a membrane; BFMG (*n* = 10), defects filled with the association of biomaterial and fibrin biopolymer, covered by a membrane; BFMLG (*n* = 10), defects filled with the association of biomaterial and fibrin biopolymer, covered by a membrane and PBMT (Figure 7).

### 4.5. Surgical Procedure

The surgery was performed at the Central Bioterium of the University of Marília (Unimar) where the rats were previously weighed and then anesthetized with intramuscular injection of tiletamine hydrochloride associated with zolazepam 10 mg/kg IM, (Telazol^®^; Fort Dodge Animal Health, Overland Park, KA, USA).

Trichotomy was performed in the frontoparietal region and disinfection with 10% polyvidone-iodine (Povidine^®^, Vic Pharma, Taquaritinga, São Paulo, Brazil). Next, a semilunar incision of four centimeters was made with a No. 15 carbon steel scalpel blade (Embramax^®^, São Paulo, Brazil) in the integument, and the periosteum was carefully detached with the aid of the syndesmotome and folded together with the other tissues, exposing the external surface of the parietal bones. A 5.0 mm diameter circular osteotomy was performed in the center of the parietal bones with the aid of a trephine drill (Neodent^®^, Curitiba, Paraná, Brazil) adapted to the electric contra-angle (Driller^®^, São Paulo, Brazil) coupled to an electric micromotor (Driller^®^, São Paulo, Brazil), at low speed (1500 rpm), under constant and abundant sterile saline irrigation (0.9% saline).

In the BMG animals, the defects were filled with 0.015 g of biomaterial mixed with blood clot. In the animals of the BFMG and BFMLG groups, the defects were filled with the association of 0.015 g of the biomaterial and 40 µL of the polymerized fibrin biopolymer (Figure 7). Next, the membrane, hydrated in 0.9% saline solution, was carefully implanted over the defects, exceeding the limits of the edges by 2.5 mm. The periosteum and integument were repositioned and sutured with 5-0 nylon (Mononylon^®^, Johnson & Johnson Company, Somerville, New Jersey, USA) and 4-0 silk (Ethicon^®^, Johnson & Johnson Company, Somerville, New Jersey, USA), respectively. The region was carefully cleaned with 2% chlorhexidine (Riohex^®^, Farmacêutica Rioquímica, São José do Rio Preto, São Paulo, Brazil).

Immediately after the surgical procedure, the animals received intramuscular injections in a single dose of 0.2 mL/kg enrofloxacin antibiotic (Flotril^®^ 2.5%, Schering-Plow, Rio de Janeiro, Brazil) and dipyrone 0.06 mL/kg (Analgex V^®^ Agener União, São Paulo, Brazil). In addition, 200 mg/kg of paracetamol (Medley^®^, São Paulo, Brazil) were dissolved in the drinking water and were available to all animals until the euthanasia period. 

### 4.6. Photobiomodulation Therapy

**Figure 8:**
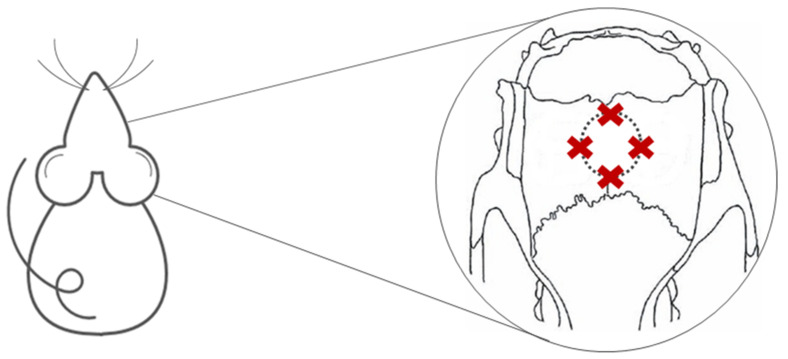
Demonstration of the four points of PBMT application at the defect margins**.**

Immediately after the surgical procedure, the GBFML animals were submitted to photobiomodulation therapy (Ibramed Laserpulse^®^, Amparo, São Paulo, Brazil) until the end of the experiment (Figure 8), 14 and 42 days, using the following protocol, previously used by our group of researchers in pre-clinical experimental studies in tissue engineering [18,25,26,49,57,76] (Table 2):

In all applications, the laser beam emissions were calibrated on the device itself and previously tested to certify the dose.

### 4.7. Sampling and Histological Procedures

After 14 and 42 days, five animals in each group were subjected to excessive doses of anesthetic, 2.5% sodium thiopental (Thiopentax^®^, Cristália, Produtos Químicos Farmacêuticos, São Paulo, Brazil) intraperitoneally, and an intramuscular injection of 1% lidocaine hydrochloride (Blau^®^ Farmacêutica SA, São Paulo, Brazil).

The calvaria of each animal were removed preserving the supraperiosteal soft tissues and fixed in a 10% buffered formalin solution and later were destined for computerized microtomography analysis.

### 4.8. Micro-CT

After fixing the bone fragments, the pieces were subjected to an X-ray beam scan in the SkyScan 1174v2 computerized microtomograph (Bruker-microCT^®^, Kontich, Belgium) of the Discipline of Endodontics, Bauru School of Dentistry (University of São Paulo). The X-ray beam sources (Cone-Beam) were operated at 50 kV, 800 μA, using a Cu + Al filter and rotated through 360°, with a 0.5 rotation step and an isotropic resolution of 19.6 µm.

The images were reconstructed using the NReconTM v.1.6.8.0 program (SkyScan^®^, 2011, Bruker-microCT), with the same reconstruction parameters for all samples. Next, the reconstructed images were realigned with the DataViewer^®^ 1.4.4.0 software resulting in two-dimensional transaxial and sagittal images with 16-bit gray scale resolution.

### 4.9. Histotechnical Processing

The pieces were then washed in running water for 24 h and subjected to demineralization in 10% EDTA (4.13% tritiplex^®^ III, Merck KGaA, Hessen, Germany and 0.44% sodium hydroxide^®^, Labsynth, São Paulo, Brazil), with weekly changes of the solution for a period of approximately six weeks, carried out at the Anatomy Laboratory of Bauru School of Dentistry (University of São Paulo).

The bone fragments collected were submitted to standard histological processing and included in a HistosecTM (Merck KGaA^®^, Darmstadt, Germany) at the Histology Laboratory of the University of Marília (Unimar). Semi-serial coronal slices with 5 µm thicknesses were made, prioritizing the center of the defect, and stained with hematoxylin-eosin, Masson’s trichrome, and picrosirius-red.

The defect images were obtained using the Leica DFC 310FX high resolution digital camera (Leica^®^, Microsystems, Wetzlar, Germany) connected to the Leica DM IRBE inverted laser microscope and LAS 4.0.0 capture system (Leica^®^, Microsystems, Heerbrugg, Switzerland).

Each type of fiber by color was analyzed using the Axio Vision Rel. 4.8 Ink image software (Carl Zeiss^®^ MicroImaging GmbH, Jena, Germany). The interlaced bone was recognized for its random and unorganized fibrillar pattern, usually with polarization colors ranging from red/orange to light green/yellow, depending on the width of the fiber.

### 4.10. Histomorphological and Histomorphometric Analyses

For the histomorphological description of the areas of the bone defect in all animals, the central region and the border were considered to evaluate the incorporation of the biomaterial in the recipient bed, the formation of granulation tissue, infiltration of inflammatory and blood cells and tissue formation primary bone (immature bone) and secondary bone (lamellar bone—bone maturation). For this, four semi-serial sections of the surgical bed were evaluated by slide for each animal.

For histomorphometric analysis, blades stained with Hematoxylin and Eosin were observed under the Olympus BX50 light microscope (Olympus^®^ Corporation, Tokyo, Japan) and the photographs captured in 4x lens with the digital camera attached (Olympus^®^ DP 71, Tokyo, Japan).

Quantitative analysis was performed on the computer (Pentium^®^ Core 2 Duo processor; Intel Corporation, Santa Clara, CA, USA) using the Axio Vision Rel. 4.8 Ink image software program (Carl Zeiss^®^ Carl Zeiss MicroImaging GmbH, Jena, Germany).

From the semi-serial sections obtained, two more central sections of the defect were captured with a distance of 300 μm between them. The total area of the removed bone block or total area of the defect (TA) and the area of new bone formation (ANB) of each defect were determined. The percentage of newly formed bone tissue (PNB) was calculated by the following relationship: P_NB_ = A_NB_ × 100/TA [26].

### 4.11. Statistical Analysis

Statistical analysis was performed on the computer using the GraphPad Prism 8 Program (GraphPad^©^ Software 2018, San Diego, CA, USA).

To analyze the influence of time on the percentage of new bone formation within each experimental group, an “unpaired *t*-test” was used and, for comparative analysis of the percentage of new bone formed by comparing the groups within the same experimental period, an ANOVA for independent samples and the “post hoc” Tukey’s test were used.

For all analyses, *p* values < 0.05 were considered statistically significant (*n* = 5 for each period and group). Bartlett’s test was used as a test of data normality.

## 5. Conclusions

The use of photobiomodulation therapy (PBMT) on the guided bone regeneration (GBR) in defects filled with two scaffolds, the biphasic calcium phosphate and fibrin biopolymer, was evaluated in this in vivo study. The PBMT showed positive effects capable of improving and accelerating the GBR process when associated with these scaffolds. The results demonstrated a dependence on the postoperative time in the formation of new bone in all groups, with the highest density at 42 days, with the presence of secondary bone tissue, thick collagen fibers, and arranged in concentric layers. This experimental protocol demonstrates relevance in tissue bioengineering, with the use of PBMT in the association of alloplastic ceramics and bovine biological membrane already being used in clinical bone regeneration procedures, and fibrin biopolymer is in phase II/III of clinical tests, whose preclinical tests in several areas have demonstrated its translational potential.

## Figures and Tables

**Table 1:** Percentage of new bone formation in each group in the two experimental periods (14 and 42 days). BMG—defects filled with HA/β-TCP covered by a bovine biological membrane; BFMG defects filled with HA/β-TCP and heterologous fibrin biopolymer covered with bovine biological membrane; BFMLG defects filled with HA/β-TCP and heterologous fibrin biopolymer covered with bovine biological membrane and PBMT.

	14 Days	42 Days	*p* Value
BMG	4.51 ± 0.44aA	7.11 ± 0.44bA	*p* < 0.0001
BFMG	7.35 ± 0.66aB	9.41 ± 0,84bB	*p* = 0.0026
BFMLG	10.12 ± 0.67aC	13.85 ± 0.54bC	*p* < 0.0001

Different lowercase letters (line) indicate a significant difference in each group in the two periods (unpaired *t*-test). Different capital letters (column) indicate a significant difference in the same period between groups (ANOVA and Tukey). Values defined as the mean ± standard deviation (*p* < 0.05).

**Table 2:** Protocol of photobiomodulation therapy.

Parameter	Unit/Description
Type of laser	GaAlAs (gallium-aluminum-arsenide)
Output power	30 mW
Wavelength	830 nm
Power density	258.6 mW/cm^2^
Energy density	6.2 J/cm^2^
Beam area	0.116 cm^2^
Total power	2.9 J
Beam type	Positioned perpendicular to the skull
Emission mode	Continuous
Form of application	Four points around the surgical area
Irradiation duration	24 s per point
Total time of each application	96 s
Treatment time	Immediately after surgery and three times a week until euthanasia.

## Data Availability

The data presented in this study are available on request from the corresponding author. The data are not publicly available due to they are part of a master’s thesis not yet deposited in a public repository.
